# The development of TH_2_ responses from infancy to 4 years of age and atopic sensitization in areas endemic for helminth infections

**DOI:** 10.1186/1710-1492-9-13

**Published:** 2013-04-08

**Authors:** Yenny Djuardi, Taniawati Supali, Heri Wibowo, Yvonne CM Kruize, Serge A Versteeg, Ronald van Ree, Erliyani Sartono, Maria Yazdanbakhsh

**Affiliations:** 1 Department of Parasitology, Faculty of Medicine, University of Indonesia, Salemba 6, Jakarta 10430, Indonesia; 2 Department of Parasitology, Leiden University Medical Center, Leiden, The Netherlands; 3 Department of Experimental Immunology and Department of Otorhinolaryngology, Academic Medical Center, Amsterdam University, Amsterdam, The Netherlands

**Keywords:** TH_2_, IgE, IL-5, Helminth, Atopy, Skin prick test, Children

## Abstract

**Background:**

Helminth infections and allergies are associated with TH_2_ responses. Whereas the development of TH_2_ responses and allergic disorders in pediatric populations has been examined in affluent countries, no or little data exist from low income regions of the world.

The aim of this study is to examine factors influencing the development of TH_2_ responses of children born in areas endemic for helminth infections and to relate these factors to atopic sensitization at 4 years of age.

**Methods:**

Data were collected from pregnant mothers on helminth infections, education and socioeconomic status (SES). Total IgE, IL-5 in response to mitogen, and helminth antigens were measured in children at 2, 5, 12, 24 and 48 months of age. Skin prick testing (SPT) and allergen-specific IgE were determined at 4 years of age.

**Results:**

Strong TH_2_ responses were seen at 5 months of age and increased with time. Although maternal filarial infection was associated with helminth-antigen specific TH_2_ responses, it was low maternal education or SES but not helminth infection, which was associated with the development of high total IgE and PHA-induced IL-5. At 4 years of age when allergen reactivity was assessed by SPT, the high general TH_2_ responses did not translate into higher prevalence of SPT. The risk factor for SPT reactivity was low maternal education which decreased the risk of SPT positivity to allergens (adjusted OR, 0.32; 95% CI, 0.12 – 0.87) independently of maternal filarial infection which tended to reduce the child’s risk for being SPT positive (adjusted OR, 0.35; 95% CI, 0.07 – 1.70).

**Conclusions:**

In areas endemic for helminths, potent TH_2_ responses were seen early in life, but did not translate into a higher SPT reactivity to allergens. Therefore, in many parts of the world TH_2_ responses in general and IgE in particular cannot be used for diagnosis of allergic diseases.

## Background

Pregnancy and early childhood are critical periods in which the inherited immune system of a child is shaped by the environment both in utero and soon after birth. This in turn is thought to determine the disease outcome in older age. Previous studies have shown that the development of atopy can be linked to certain immunological patterns seen in cord blood or in peripheral blood during infancy. Several birth cohort studies such as in United Kingdom and Finland, revealed that higher cord total IgE levels were associated with atopic manifestations at 4, 10, or even at 20 years of age [1,2], while in another large birth cohort study in UK atopic children at 5 years of age had higher total IgE at 12 months [3]. In a small birth cohort study in Australia, children with atopy at 2 years of age already exhibited a decreased production of general adaptive T helper (Th)1 response at birth, followed by increasing allergen-specific-TH_2_ from 6 till 18 months of age, indicating that immune responses can be already skewed early in childhood [4]. Another birth cohort study in United States showed that the levels of general adaptive TH_2_ cytokines at 3 months and 1 year of age was positively associated with total IgE at 1, 2, 3 and 5 years of age [5]. All these studies demonstrated that any disturbance of immune responses which can translate into allergies in childhood seems to originate from very early life or in the first year of life. However, such important longitudinal studies have been conducted in affluent countries only. Given the alarming increase of allergic disorders in urban centers of low to middle income countries [6], it would be important to understand the relationship between TH_2_ responses and allergic disorders in these countries where helminth infections can be highly prevalent.

Helminth parasites, like allergies, have been shown to be associated with TH_2_-skewed immune responses characterized by the increased production of TH_2_ cytokines (IL-4, IL-5, IL-13), polyclonal and specific IgE responses, and eosinophilia [7]. Interestingly, a number of studies show that the presence of helminth infections is inversely associated with atopy as defined by skin sensitization [8], while association with allergic diseases or asthma have been less convincing and if anything, helminths seem to be associated with increased IgE responses to environmental allergens [9]. However, in studies conducted in developing countries, the presence of IgE antibodies to allergens, has not always been a reliable predictor of skin prick test positivity or allergic diseases, especially in areas where helminth infections were prevalent [10]. Thus, many children with high total IgE or positive allergen-specific IgE had negative skin test reactivity [9] or reported symptoms of allergy [11]. In this context, socioeconomic factors can play an important role as has been shown by the multicenter ISAAC study in affluent and less-affluent countries where the positive association between atopic sensitization and atopic wheeze was weaker in countries with low gross national income per capita [12]. Moreover it has been suggested that the result might apply to the association between IgE and skin test reactivity [13]. These studies were mainly cross-sectional and conducted among schoolchildren or adults. Little is known about the development of TH_2_ responses at earlier time points in these less affluent countries, where helminth infections might be highly prevalent, and the relationship with allergic outcomes.

To start addressing this, we have examined the development of TH_2_ responses and influencing factors in early life of children living in an area in Indonesia where helminth infections are prevalent. We hypothesize that early exposure to certain maternal environmental factors including helminth infection during pregnancy will affect the development of TH_2_ responses. Furthermore, we have related these factors to atopic sensitizations at 4 years of age.

## Methods

### Study population and parasitological examination

**Figure 1:**
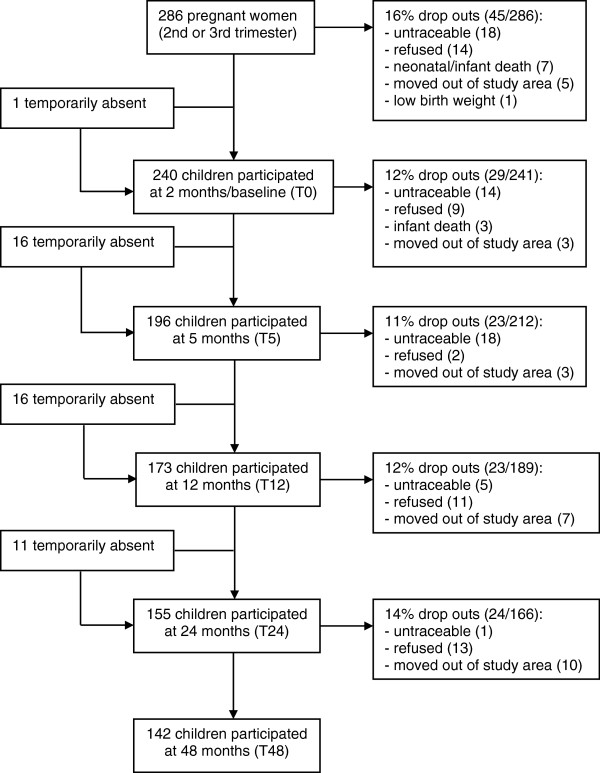
**The flow diagram of the cohort study.** Drop outs imply that the participants have no data from this time point onward. Temporarily absent implies that the participants have no data at this time point but have data available at other time point.

The participants for this cohort study consisted of pregnant mothers and their subsequently born children living in two adjacent villages, which were endemic for filarial (*Wuchereria bancrofti*) and soil-transmitted helminth (STH) infections, in Bekasi District, Indonesia, as described previously [14]. Between 2002 and 2004, pregnant mothers who came to collaborating midwives for their prenatal care at the second or third trimester were enrolled. The children born full-term and healthy and whose mothers agreed to continue participation were followed up during house-to-house visits at 2 months (T0); 5 months (T5); 1 year (T12); 2 years (T24); and 4 years (T48) of age. A total of 84.3% (241/286) of children of enrolled mothers who continued to participate had immunological data available at least at 1 time point. The flow diagram of the study is shown in Figure 1. The study was approved by the Ethics Committee of Faculty of Medicine, University of Indonesia.

Maternal filarial infection was determined by detection of circulating antigens in blood using immuno-chromatographic test (ICT) as described by the manufacturer (Binax, Scarborough, ME, USA). Stool samples from mothers and children at 4 years of age were examined for the presence of STH eggs by lugol staining and microscopy, and positives were treated with single dose of albendazole 400 mg.

Data on socioeconomic status (SES) factors were collected via questionnaire during recruitment of pregnant women. Each factor was given a score: type of house wall (bamboo/wood/mixed with brick = 0, brick = 1), water supply (well = 0, pump/pipe = 1) and cooking fuel (wood = 0, kerosene = 1, gas = 2). SES is low if the sum of scores ≤ 2, and high if ≥ 3. Educational level is scored 0 if no or primary schooling and 1 for higher education.

### Preparation of adult worm antigens

*Ascaris lumbricoides* adult worms were obtained from infected individuals in Indonesia and *Brugia malayi* adult worms were recovered from infected jirds. The method for antigen preparation has been described in a previous study [15].

### Whole blood culture and cytokine measurement

As previously described [16], whole blood was diluted 1:10 in RPMI 1640 medium (Invitrogen, Breda, The Netherlands) before stimulation with phytohaemagglutinin (PHA; 2 μg/mL; Wellcome Diagnostics, Dartford, UK), *B. malayi* adult crude antigen (BmA; 12.5 μg/mL), *A. lumbricoides* adult crude antigen (20 μg/mL) or medium only. After six day culture at 37°C and 5% CO_2_, supernatants were collected and frozen (−20°C) until cytokine measurement.

IL-5 in supernatant was measured by ELISA as described elsewhere [17] and with a detection limit of 2 pg/mL. All values below detection limit were given half of the detection limit. Between two and seven subjects within each time point were excluded from the entire IL-5 analysis due to very low response to mitogen (< 10 pg/mL).

### Total IgE measurement

Total IgE levels in plasma of mothers and children at all time points were measured using ELISA, adapted from the previous study [18].

### Specific IgE and skin prick testing (SPT)

Children at 4 years of age were skin tested against house dust mite (HDM, *Dermatophagoides pteronyssinus*), peanut, egg, shrimp, histamine dihydrochloride 10 mg/mL as positive control, allergen diluent as negative control (kindly provided by Paul van Rijn, HAL Allergy Laboratories, Leiden, The Netherlands) and german cockroach (*Blatella germanica*; Lofarma, Milan, Italy). The tests were performed using SPT lancets (Stallergens SA, Antony, France) and were assessed at 15 min. The result was considered positive if mean diameter of wheal (the sum of longest diameter to the perpendicular diameter/2), was at least 3 mm.

Specific IgE (sIgE) levels from pregnant women and children at 4 years of age were measured against two aeroallergens, HDM and german cockroach by ImmunoCAP® (Thermo Fisher Scientific, Uppsala, Sweden). The two allergens for sIgE measurement were chosen based on the most prevalent SPT positivity. Individuals were considered sensitized if the levels of sIgE were ≥ 0.35 kU/L.

### Statistical analysis

Total IgE levels were normalized with log-transformation and comparisons between baseline and other time points were analysed using Student t-tests. For child’s IL-5 responses which could not be normalized with log-transformation, Mann-U Whitney tests were used. Bonferroni’s correction was applied for multiple comparisons. Mean levels were expressed as geometric means with 95% confidence interval (CI), while median levels were presented with interquartile range (IQR).

To be able to analyze all children in a longitudinal fashion including those with missing data at a given time point, a linear mixed model for log total IgE was performed to include individuals as the random effect. Maternal helminth infections, village of residence, SES, education, time variable and child’s baseline immunological data were treated as fixed effects. For the longitudinal analysis of binary outcomes of IL-5 (with median value as the cut-off), General Estimating Equation (GEE) analysis was performed to include time as the within subject variable and other factors as covariates. In addition to adjusting for the covariates in univariate models, all multivariate models were also adjusted for child’s gender. An interaction term with time was tested only for factors that have effects on an outcome variable over time.

To explore the association between maternal factors and child’s atopic sensitization (SPT or allergen-specific IgE), univariate and multivariate logistic regression analyses were performed. *P* values were two-sided and were considered significant if < 0.05. All statistical analyses were done in SPSS 17.0 (SPSS Inc., Chicago, IL, USA).

## Results

### Maternal and child’s baseline characteristics

The baseline characteristics of mothers and children are shown in Table 1. Sixty four percent (153/240) of pregnant women had a low level of education and 43% (99/236) were classified as having low socioeconomic status (SES). Although low SES was more likely to be found in mothers with low education (49% in low-educated versus 29% in high-educated mothers), the overlap was only partial and therefore these two variables had to be considered separately. The percentage of pregnant women infected with filarial parasites was 19% (45/241) and with intestinal helminths was 31% (68/217). The loss to follow up at the end of the study was 41% (99/241). There were no significant differences in gender, maternal helminth infections, education and SES between those who remained in the study and the group that was lost to follow up, except for village of residence (Jati Karya village: 63% vs 43%, respectively, Chi-square test: p = 0.002). At four years of age, 10 out of 126 children (8%) were infected with soil transmitted helminths (STH) and since the prevalence was low, it was not considered in data analysis.

**Table 1:** Baseline characteristics of mothers and children participating at the baseline (T0)

**Participants**	**Characteristics**	**n/N (%)**
Pregnant mothers	Village of residence	
	Jati Sampurna	109/241 (45)
	Jati Karya	132/241 (55)
	Filarial infections	45/241 (19)
	STH infections	68/217 (31)
	*Ascaris lumbricoides*	33/217 (15)
	Hookworms	29/217 (13)
	*Trichuris trichiura*	13/217 (6)
	Socioeconomic status	
	Low	99/236 (42)
	High	137/236 (58)
	Educational level	
	Low	153/240 (64)
	High	87/240 (36)
Children	Gender	
	Boys	114/230 (50)
	Girls	116/230 (50)
	STH infections at 4 yrs	10/126 (8)
	* Ascaris lumbricoides*	8/126 (6)
	* Trichuris trichiura*	5/126 (4)

STH, soil-transmitted helminth.

### The development of child’s general and specific TH_2_-type responses

**Figure 2:**
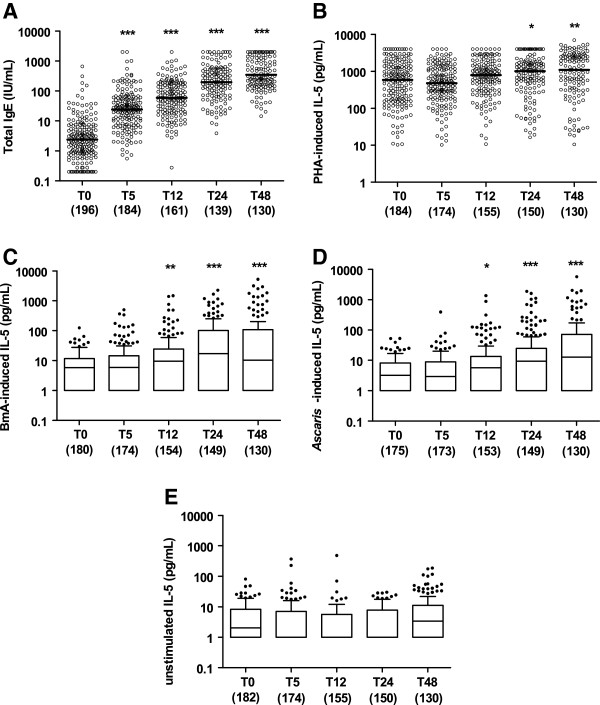
**Longitudinal pattern of child’s general and specific TH**_**2**_**-type humoral and cellular immune responses.** The levels of child’s total IgE (**A**), PHA-induced IL-5 (**B**), BmA-induced IL-5 (**C**), *Ascaris*-induced IL-5 (**D**) and unstimulated IL-5 (**E**) of children at the age of 2 months (T0), 5 months (T5), 12 months (T12), 24 months (T24) and 48 months (T48). The horizontal lines in the bars represent the median, with the lower 25% and upper 75% percentiles and extended whiskers the 10% and 90% percentiles. Comparisons were made between the baseline (T0) and other time points: *p < 0.05; **0.001 < p < 0.01, ***p < 0.001.

The pattern of child’s total IgE production was markedly increased between T0 and other time points (*P* < 0.001; Figure 2A). Geometric mean (GM; 95% CI) of total IgE was 2.4 (1.9 – 3.0) IU/mL at 2 months, 24.0 (19.3 – 29.8) IU/mL at 5 months, 59.3 (47.7 – 73.8) IU/mL at 1 year, 196.8 (156.1 – 248.1) IU/mL at 2 years, and 345.9 (280.6 – 426.4) IU/mL at 4 years of age. The increasing levels of general TH_2_-type cytokine responses, represented by mitogen-stimulated IL-5 production, were seen in particular at T24 and T48 (Figure 2B).

In response to helminth antigens (BmA and *Ascaris*), IL-5 production increased significantly to reach a plateau at 4 years of age (Figure 2C and D). Spontaneous IL-5 production was low and did not increase significantly over the same time period (Figure 2E).

### Association between maternal factors and development of child’s TH_2_-type responses

**Figure 3:**
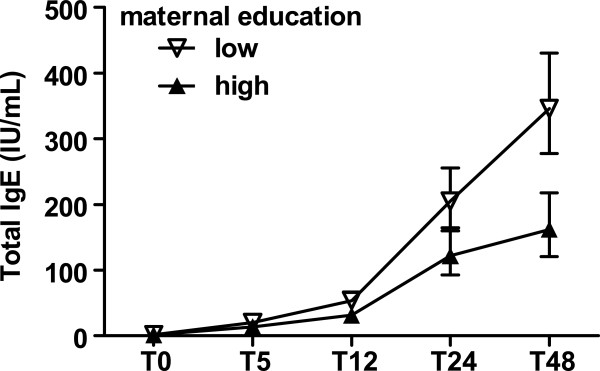
**Total IgE of children as a function of maternal educational status.** Open triangle are children born to low-educated mothers, closed triangle are children born to high-educated mothers. Error bars represent geometric means and 95% confidence intervals of child’s total IgE, after adjustment for maternal helminth infection, village of residence, maternal education and socioeconomic status, child gender.

In the univariate linear model, village of residence, low maternal education and low SES were associated with higher total IgE levels (Table 2). In the multivariate model, maternal education was the strongest factor inversely associated with child’s total IgE (adjusted estimate, 0.15; 95% CI, 0.05 – 0.25), followed by maternal SES (adjusted estimate, 0.12; 95% CI, 0.02 – 0.21) and village of residence (adjusted estimate, -0.21; 95% CI, -0.41 – (−0.01)). Figure 3 shows the difference in total IgE levels between children with low and high maternal educational level, where starting from 1 year of age, children with low-educated mothers had higher total IgE levels compared to those with high-educated mothers. This change with time is indicated by the significant interaction between maternal education and time (Table 2).

**Table 2:** Association between maternal factors and child total IgE or IL-5 over time

**Variables**	**Total IgE**		**IL5 PHA**		**IL5 BmA**		**IL5 Asc**	
	**crude β (95%CI)**	adj β* (95%CI)	**crude OR (95%CI)**	adj OR* (95%CI)	**crude OR (95%CI)**	adj OR* (95%CI)	**crude OR (95%CI)**	adj OR* (95%CI)
**Maternal filaria**								
No	Ref	Ref	Ref	Ref	Ref	Ref	Ref	Ref
Yes	0.06	0.04	1.17	1.04	1.67	1.64	1.68	1.69
	(−0.04, 0.17)	(−0.08, 0.16)	(0.87, 1.57)	(0.76, 1.41)	(1.20, 2.33)	(1.14, 2.36)	(1.15, 2.46)	(1.11, 2.58)
*P* value	0.24	0.51	0.30	0.81	**0.003**	**0.008**	**0.007**	**0.01**
*P*-int**	NT	NT	NT	NT	**0.049**	**0.04**	0.52	0.50
**Maternal STHs**								
No	Ref	Ref	Ref	Ref	Ref	Ref	Ref	Ref
Yes	0.08	0.05	0.87	0.83	1.00	0.91	1.07	0.97
	(−0.02, 0.18)	(−0.05, 0.15)	(0.65, 1.18)	(0.61, 1.13)	(0.74, 1.36)	(0.67, 1.23)	(0.76, 1.50)	(0.69, 1.38)
*P* value	0.12	0.32	0.38	0.23	1.00	0.54	0.69	0.89
*P*-int**	NT	NT	NT	NT	NT	NT	NT	NT
**Village of residence**								
Jati Sampurna	Ref	Ref	Ref	Ref	Ref	Ref	Ref	Ref
JatiKarya	−0.13	−0.21	1.10	1.11	1.08	0.97	0.93	0.84
	(−0.93, 0.67)	(−0.41, -0.01)	(0.83, 1.45)	(0.83, 1.49)	(0.81, 1.44)	(0.70, 1.34)	(0.67, 1.28)	(0.60, 1.19)
*P* value	0.29	**0.04**	0.50	0.47	0.62	0.87	0.65	0.33
*P*-int**	NT	0.30	NT	NT	NT	NT	NT	NT
**Socioeconomic status**								
High	Ref	Ref	Ref	Ref	Ref	Ref	Ref	Ref
Low	0.13	0.12	1.44	1.47	1.20	1.16	1.39	1.20
	(0.04, 0.22)	(0.02, 0.21)	(1.10, 1.87)	(1.11, 1.95)	(0.89, 1.61)	(0.86, 1.58)	(1.02, 1.91)	(0.86, 1.68)
*P* value	**0.006**	**0.02**	**0.007**	**0.007**	0.23	0.33	**0.04**	0.28
*P*-int**	0.20	0.11	0.61	0.61	NT	NT	0.50	NT
**Maternal education**								
High	Ref	Ref	Ref	Ref	Ref	Ref	Ref	Ref
Low	0.16	0.15	1.08	0.93	1.02	0.87	1.43	1.22
	(0.07, 0.25)	(0.05, 0.25)	(0.81, 1.43)	(0.67, 1.27)	(0.75, 1.40)	(0.63, 1.21)	(1.03, 1.98)	(0.88, 1.70)
*P* value	**0.001**	**0.003**	0.60	0.63	0.89	0.42	**0.03**	0.23
*P*-int**	0.07	**0.03**	NT	NT	NT	NT	**0.03**	NT

*Adjusted for other factors including child gender.

** *P*-int: *P* value on the interaction between time and the corresponding variable.

*P* value in bold if significant (< 0.05).

Adj: adjusted, OR: odds ratio, Ref: reference, NT: Not tested.

STH: soil-transmitted helminth, PHA: phytohemagglutinin, BmA: *Brugia malayi* adult worm antigen, Asc: *Ascaris* adult worm antigen.

The child’s general TH_2_-type cytokine production (PHA-induced IL-5) up to 4 years of age was only affected by maternal SES (adjusted odds ratio [adj OR] 1.47; 95% CI, 1.11 – 1.95; Table 2). Regarding helminth specific TH_2_-cytokine responses, there were positive associations between maternal filarial infection and IL-5 induced by BmA or *Ascaris* antigen, that remained significant after adjusting for village of residence, maternal education and SES (adj OR, 1.64; 95% CI, 1.14 – 2.36 and adj OR, 1.69; 95% CI, 1.11 – 2.58, respectively).

### Factors associated with child atopy

Of 132 children having SPT, there were 2 children with positive SPT for negative control and 3 children with no histamine wheal; these children were excluded from further analysis. Of 127 children with available SPT results, 24 children (19%) were positive for any allergen tested. There were 21 children (16%) positive for cockroach and 9 children (7%) positive for house dust mite (HDM), with a total of 22 children (17%) positive for either or both aeroallergens. Among the skin test reactivity for food allergens, three children (2%) were sensitized to shrimp, one child (0.8%) to peanut and none were sensitized to egg. From those children with available specific IgE (sIgE) measurements, 58% (61/105) of children were positive for HDM with median of 0.93 (interquartile range [IQR], 0.10 – 3.21) kU/L and 55% (58/106) were positive for cockroach with median of 0.60 (IQR, 0.09 – 1.88) kU/L. In total, 64% (68/107) of the children had positive sIgE to either HDM and/or cockroach.

To see whether SPT results correlated well with allergen-specific or total IgE measured at the same age, the IgE levels were compared between SPT-positive and SPT-negative children. No significant association was found between sIgE positivity and SPT. From a total of 93 children with available SPT and sIgE results, all 17 children with positive SPT, either to HDM or cockroach, had sIgE levels above 0.35 kU/L; while about half of children with negative SPT (41 out of 76) still had sIgE levels above 0.35 kU/L. We found no significant associations between total IgE and SPT reactivity, where SPT-positive children (n = 19; GM, 396.4; 95% CI, 208.4 – 753.9 IU/mL) had similar levels of total IgE compared to SPT-negative children (n = 95; GM, 330.3; 95% CI, 259.0 – 421.1 IU/mL).

Next we asked whether maternal factors would affect the child atopy at 4 years of age, a time point when SPT and sIgE to allergens were measured. Low maternal education was significantly associated with lower risk of child SPT positivity and remained significant in the multivariate analysis (adj OR, 0.32; 95% CI, 0.12 – 0.87; Table 3). Negative trends were seen for an independent association between maternal filarial infection during pregnancy and child SPT (OR, 0.27; 95% CI, 0.06 – 1.25); however when adjusted for other factors, the confidence interval widened (Table 3). In terms of child sIgE, no significant association was found with maternal education or filarial infection. In contrast to maternal education, socioeconomic status was not significantly associated with any of the atopic markers.

**Table 3:** Association between maternal factors and child’s atopy at 4 years of age

**Variables associated with child’s skin prick test positivity**	**n (%) N = 126**	**crude OR (95%CI)**	***P *value**	adj OR (95%CI)*	***P *value**
Maternal filaria	30 (24)	0.27 (0.06, 1.25)	0.09	0.35 (0.07, 1.70)	0.20
Village of residence (Jati Karya)	84 (67)	0.69 (0.27, 1.78)	0.44	0.84 (0.31, 2.28)	0.74
Socioeconomic status (low)	59 (47)	0.75 (0.29, 1.90)	0.54	1.02 (0.37, 2.82)	0.97
Maternal education (low)	87 (69)	0.29 (0.11, 0.74)	**0.01**	0.32 (0.12, 0.87)	**0.02**
**Variables associated with child’s allergen-specific IgE**^**§**^	**n (%) N = 105**	**crude OR (95%CI)**	***P *value**	adj OR (95%CI)*	***P *value**
Maternal filaria	25 (24)	0.43 (0.17, 1.07)	0.07	0.43 (0.16, 1.15)	0.09
Village of residence (Jati Karya)	60 (57)	0.75 (0.33, 1.66)	0.47	0.83 (0.35, 1.97)	0.68
Socioeconomic status (low)	44 (42)	1.17 (0.52, 2.63)	0.70	1.41 (0.59, 3.32)	0.44
Maternal education (low)	69 (66)	0.68 (0.29, 1.59)	0.37	0.75 (0.30, 1.87)	0.54

*Adjusted for other factors including child’s gender.

^§^Allergen-specific IgE as the binary outcome using cut-off 0.35 kU/L.

*P* value in bold if significant (< 0.05).

Adj: adjusted, OR: odds ratio.

## Discussion

This follow-up study from birth to 4 years of age was conducted in an area endemic for helminth infections, and demonstrated development of strong TH_2_ type responses. Here, the total IgE levels already present at 5 months of age were three times higher than those in the United States [19] and at 4 years of age the levels were at least ten times higher than those in the Netherlands [20]. These strong TH_2_ responses early in life, did not translate into proportionally higher prevalence of SPT to allergens at 4 years of age which at 19% was similar or even lower than the prevalence in affluent countries: 23% at the age of 3 yrs in Denmark [21] and 19.6% at the age of 4 yrs in UK [22]. Surprisingly, the children from our study had two to four times higher prevalence of aeroallergen-sIgE compared to children of affluent countries at the same age [23,24]. To understand this we have assessed factors that affected the development of general TH_2_ responses as well as atopic outcomes at 4 years of age.

While maternal filarial infection was strongly associated with child’s helminth-specific TH_2_ responses, it did not appear to play an important role in the development of general TH_2_ responses. This indicates that maternal parasitic helminth status was not the strongest factor determining the TH_2_ polarization in these young Indonesian children. Indeed further analysis revealed that maternal education and or socioeconomic status had a significant influence on the development of total IgE responses. Children born to mothers with low SES or low education showed stronger development of total IgE responses over time than children born to mothers with high SES or high education. Interestingly at 4 years of age, children born to low-educated mothers had less chance of skin test positivity while maternal filarial infection had a tendency, independently of education level, to reduce the chance of a child being SPT positive.

The increasing total IgE in children up to four years of age in our study was in line with a cross-sectional study in young Ethiopian children where it was shown that IgE levels were 16 to 20 times higher compared to the levels in healthy Swedish children [25]. Recent cohort studies which were mostly conducted in children from affluent countries have studied total IgE and related it to allergic outcomes. It was shown that increasing levels of IgE were associated with atopic sensitization (defined as positive SPT) [3] or allergy symptoms [26]. However, in our study the elevated total IgE did not seem to translate into high prevalence of SPT, the reason being that the total IgE measured in our study reflects largely IgE whose specificity remains elusive and is possibly clinically irrelevant. The levels of total IgE, however, can be very different between countries and races, which is partly explained by genetic factors [27]; although other factors can play a role too. Often helminths have been shown to influence total IgE [25,28,29]. In our study population, higher maternal education or socioeconomic status led to lower total IgE in a child. We consider these maternal factors as proxies for specific factors that influence child’s immune development. In previous epidemiological studies, higher maternal education and SES have been associated with better hygiene practices [30] which in turn could decrease exposure to microorganisms and parasites [31,32]. Although the prevalence of child’s intestinal helminth infection at 4 years of age was low (8%), it is possible that past infection, exposure to helminth eggs without established infection or infection by other parasites induced TH_2_-type responses. In addition, other orofecal pathogens which were not measured in this study have been associated with decreased atopy in infected individuals [33,34]. Besides hygiene, maternal education and SES can also be correlated with feeding practices (quality of nutrition and timing of first solid food introduction), maternal stress, antibiotics prescription, exposure to endotoxin and pollutants. All these factors can have long lasting impacts on a child’s atopic outcomes [35].

Our study showed that maternal filarial infection clearly led to helminth antigen specific TH_2_ responses. Children can be sensitized to pathogen-derived antigens or allergens in utero [19,36-39]. In our study, maternal filarial infection affected the development of child’s helminth-specific TH_2_ responses indicating in utero sensitization of child’s immune cells to filarial parasite antigens. Our results are in line with previous studies in areas endemic for filariasis showing that T cells and B cells of the newborns were capable of producing helminth-specific TH_2_ cytokines and specific IgE antibodies [40,41].

While on the one hand maternal filarial infection clearly affected child’s helminth-specific TH_2_ responses, on the other hand, it showed a tendency to decrease child’s skin reactivity and specific IgE production to allergens. Thus, early exposure to helminths might by stimulating helminth-antigen specific TH_2_-type responses, attenuate and deviate from mounting a TH_2_ response to allergens. Interestingly, exposure to filarial infection in earlier time points in these young children with a tendency for decreased skin reactivity does not appear different from what is seen in older subjects where increasing prevalence of filarial infection was paralleled with decreasing skin reactivity to aeroallergens [42]. However, the difference seems to lie in the effect of helminth infection on allergen-specific IgE. In many studies so far, populations in areas where helminths are endemic bear high allergen-specific IgE [9,43,44]. Yet, in the current study we see that in early life, maternal filarial infection which is associated with helminth-antigen specific TH_2_ responses, is associated with lower allergen-specific IgE. Whether the allergen-specific IgE induced during early childhood represents more of a “real” sensitization to aeroallergens compared to those at older age reflecting more of cross-reactivity between helminth- and allergen-specific IgE [45] needs to be investigated further. The possible effect of helminth infections during pregnancy on child’s allergic disorders is supported by a randomized-controlled study in Uganda showing that praziquantel treatment of pregnant mother infected with *Schistosoma mansoni* increased the risk of eczema in their infants [46]. Moreover the group also found that eczema was positively associated with allergen skin test result in infants, although they did not look into the direct association between the effect of maternal treatment and child’s SPT.

The percentage of children who were lost to follow up at the end of the study was considered high (41%). Since the participants were similar to the non-participants with only difference in the village of residence, we considered the possibility of bias minimal, as we have included village as a factor in the multivariate models. For child atopic outcomes, since the number of tested children decreased at the end of study and that the skin test reactivity prevalence was low, these factors could weaken the power of analysis. However as the negative trend of association between maternal filarial infection and child’s skin test reactivity was also shown for allergen-specific IgE, this could indeed mean that exposure to maternal filarial infection affected child’s atopy. For more confirmation on this finding, a similar cohort study with larger sample size would be needed by taking into account the prevalence of maternal helminth infection and child’s skin test reactivity. With the limited number of potential confounders measured in our study and thus included in the analysis, it is possible that other uncontrolled confounders present during pregnancy and after birth could still affect the development of child’s Th2-type immune responses or atopy. Among these, family atopy, household crowding, duration of breastfeeding and pet ownership.

## Conclusions

In summary, this longitudinal study shows that young children born in areas endemic for helminth infections develop strong TH_2_ responses that increase with age at least up to 4 years of age, while the prevalence of SPT is not increased proportionally. Different maternal environmental factors determined by SES and education affect child’s TH_2_ responses. The challenge for the future is to delineate what precise factors mediate the effect of maternal SES and education on general TH_2_ responses as well as SPT to allergens. In addition, further studies are needed to address whether the protection against atopy will translate into less asthma and allergic disorders in later life.

## Consent

Written informed consent was obtained from the child’s guardian/parent/next in keen for publication of this report and any accompanying images.

## Abbreviations

TH2: T helper 2; sIg: specific Immunoglobulin; IL: Interleukin; PHA: Phytohaemagglutinin; BmA: *Brugia malayi* Antigen; SPT: Skin prick test; HDM: House dust mite; SES: Socioeconomic status; STH: Soil-transmitted helminth; ICT: Immuno-chromatographic test; ELISA: Enzyme-linked immunosorbent assay; ISAAC: International Study of Asthma and Allergies in Childhood; GEE: Generalized estimated equation; OR: Odds ratio; adj: Adjusted; GM: Geometric mean; CI: Confidence interval; IQR: Inter quartile range; IU: International unit; kU/L: Kilounits per liter; mg: Milligram; μg: Microgram; pg: Pictogram; mL: Milliliter; μL: Microliter; °C: Degrees celsius.

## Competing interests

All authors have declared that they have no competing interests.

## Authors’ contribution

MY conceived of the study and developed its design. YD carried out the field study, performed the statistical analyses and drafted the manuscript. TS participated in the study design and its coordination. HW participated in the study design and data collection. YCM participated in supervising the immunoassays and helped the interpretation of the results. SAV carried out the measurement of specific IgE. RvR supervised the measurement of specific IgE detection and helped to edit the manuscript. ES developed the immunoassays and helped to draft the manuscript. All authors read and approved the final manuscript.

## References

[B1] Kurukulaaratchy RJ Matthews S Arshad SH Defining childhood atopic phenotypes to investigate the association of atopic sensitization with allergic disease Allergy 2005 60 1280 1286 10.1111/j.1398-9995.2005.00890.x 16134995

[B2] Pesonen M Kallio MJ Siimes MA Elg P Bjorksten F Ranki A Cord serum immunoglobulin E as a risk factor for allergic symptoms and sensitization in children and young adults Pediatr Allergy Immunol 2009 20 12 18 10.1111/j.1399-3038.2008.00736.x 18298422

[B3] Perkin MR Strachan DP Hc W Lack G Golding J The predictive value of early life total immunoglobulin E measurement in identifying atopic children in a population-based birth cohort study Pediatr Allergy Immunol 2006 17 118 124 10.1111/j.1399-3038.2005.00364.x 16618361

[B4] Prescott SL Macaubas C Smallacombe T Holt BJ Sly PD Holt PG Development of allergen-specific T-cell memory in atopic and normal children Lancet 1999 353 196 200 10.1016/S0140-6736(98)05104-6 9923875

[B5] Rothers J Halonen M Stern DA Lohman IC Mobley S Spangenberg A Adaptive cytokine production in early life differentially predicts total IgE levels and asthma through age 5 years J Allergy Clin Immunol 2011 128 397 402 10.1016/j.jaci.2011.04.044 21683432 PMC3149723

[B6] Asher MI Montefort S Bjorksten B Lai CK Strachan DP Weiland SK Worldwide time trends in the prevalence of symptoms of asthma, allergic rhinoconjunctivitis, and eczema in childhood: ISAAC phases One and three repeat multicountry cross-sectional surveys Lancet 2006 368 733 743 10.1016/S0140-6736(06)69283-0 16935684

[B7] Yazdanbakhsh M van den Biggelaar A Maizels RM Th2 Responses without atopy: immunoregulation in chronic helminth infections and reduced allergic disease Trends Immunol 2001 22 372 377 10.1016/S1471-4906(01)01958-5 11429321

[B8] Feary J Britton J Leonardi-Bee J Atopy and current intestinal parasite infection: a systematic review and meta-analysis Allergy 2011 66 569 578 10.1111/j.1398-9995.2010.02512.x 21087217

[B9] van den Biggelaar AH Lopuhaa C van RR van der Zee JS Jans J Hoek A The prevalence of parasite infestation and house dust mite sensitization in gabonese schoolchildren Int Arch Allergy Immunol 2001 126 231 238 10.1159/000049519 11752881

[B10] Flohr C Quinnell RJ Britton J Do helminth parasites protect against atopy and allergic disease? Clin Exp Allergy 2009 39 20 32 10.1111/j.1365-2222.2008.03134.x 19128351

[B11] Hogewoning AA Larbi IA Addo HA Amoah AS Boakye D Hartgers F Allergic characteristics of urban schoolchildren with atopic eczema in ghana J Eur Acad Dermatol Venereol 2010 24 1406 1412 10.1111/j.1468-3083.2010.03655.x 20456550

[B12] Weinmayr G Weiland SK Bjorksten B Brunekreef B Buchele G Cookson WO Atopic sensitization and the international variation of asthma symptom prevalence in children Am J Respir Crit Care Med 2007 176 565 574 10.1164/rccm.200607-994OC 17575099

[B13] Obeng BB Hartgers F Boakye D Yazdanbakhsh M Out of africa: what can be learned from the studies of allergic disorders in africa and africans? Curr Opin Allergy Clin Immunol 2008 8 391 397 10.1097/ACI.0b013e32830ebb70 18769190

[B14] Djuardi Y Wibowo H Supali T Ariawan I Bredius RG Yazdanbakhsh M Determinants of the relationship between cytokine production in pregnant women and their infants PLoS One 2009 4 e7711 10.1371/journal.pone.0007711 19898617 PMC2768784

[B15] Yazdanbakhsh M Paxton WA Kruize YC Sartono E Kurniawan A van het WA T cell responsiveness correlates differentially with antibody isotype levels in clinical and asymptomatic filariasis J Infect Dis 1993 167 925 931 10.1093/infdis/167.4.925 8450257

[B16] Eriksson M Sartono E Martins CL Bale C Garly ML Whittle H A comparison of ex vivo cytokine production in venous and capillary blood Clin Exp Immunol 2007 150 469 476 10.1111/j.1365-2249.2007.03515.x 17924971 PMC2219377

[B17] Grogan JL Kremsner PG Deelder AM Yazdanbakhsh M Elevated proliferation and interleukin-4 release from CD4+ cells after chemotherapy in human schistosoma haematobium infection Eur J Immunol 1996 26 1365 1370 10.1002/eji.1830260628 8647218

[B18] van den Biggelaar AH van RR Rodrigues LC Lell B Deelder AM Kremsner PG Decreased atopy in children infected with schistosoma haematobium: a role for parasite-induced interleukin-10 Lancet 2000 356 1723 1727 10.1016/S0140-6736(00)03206-2 11095260

[B19] Havstad S Wegienka G Zoratti EM Lynch SV Boushey HA Nicholas C Effect of prenatal indoor pet exposure on the trajectory of total IgE levels in early childhood J Allergy Clin Immunol 2011 128 880 885 10.1016/j.jaci.2011.06.039 21820714 PMC3185205

[B20] Reijmerink NE Kerkhof M Bottema RW Gerritsen J Stelma FF Thijs C Toll-like receptors and microbial exposure: gene-gene and gene-environment interaction in the development of atopy Eur Respir J 2011 38 833 840 10.1183/09031936.00099210 21349911

[B21] Kjaer HF Eller E Andersen KE Host A Bindslev-Jensen C The association between early sensitization patterns and subsequent allergic disease. The DARC birth cohort study Pediatr Allergy Immunol 2009 20 726 734 10.1111/j.1399-3038.2009.00862.x 19744222

[B22] Arshad SH Tariq SM Matthews S Hakim E Sensitization to common allergens and its association with allergic disorders at age 4 years: a whole population birth cohort study Pediatrics 2001 108 E33 10.1542/peds.108.2.e33 11483843

[B23] Kerkhof M Wijga A Smit HA de Jongste JC Aalberse RC Brunekreef B The effect of prenatal exposure on total IgE at birth and sensitization at twelve months and four years of age: the PIAMA study Pediatr Allergy Immunol 2005 16 10 18 10.1111/j.1399-3038.2005.00217.x 15693906

[B24] Asarnoj A Ostblom E Kull I Lilja G Pershagen G Hedlin G Sensitization to inhalant allergens between 4 and 8 years of age is a dynamic process: results from the BAMSE birth cohort Clin Exp Allergy 2008 38 1507 1513 10.1111/j.1365-2222.2008.03046.x 18644026 PMC2610395

[B25] Johansson SG Mellbin T Vahlquist B Immunoglobulin levels in ethiopian preschool children with special reference to high concentrations of immunoglobulin E (IgND) Lancet 1968 1 1118 1121 4171844 10.1016/s0140-6736(68)90187-6

[B26] Matricardi PM Bockelbrink A Gruber C Keil T Hamelmann E Wahn U Longitudinal trends of total and allergen-specific IgE throughout childhood Allergy 2009 64 1093 1098 10.1111/j.1398-9995.2009.02055.x 19630859

[B27] Grundbacher FJ Massie FS Levels of immunoglobulin G, M, a, and E at various ages in allergic and nonallergic black and white individuals J Allergy Clin Immunol 1985 75 651 658 10.1016/0091-6749(85)90089-2 4008794

[B28] Levin ME Le Souef PN Motala C Total IgE in urban black South African teenagers: the influence of atopy and helminth infection Pediatr Allergy Immunol 2008 19 449 454 10.1111/j.1399-3038.2007.00663.x 18221478

[B29] Wahyuni S Sartono E Supali T van der Zee JS Mangali A van RR Clustering of allergic outcomes within families and households in areas endemic for helminth infections Int Arch Allergy Immunol 2005 136 356 364 10.1159/000084255 15746555

[B30] Curtale F Pezzotti P Sharbini AL Al MH Ingrosso P Saad YS Knowledge, perceptions and behaviour of mothers toward intestinal helminths in upper Egypt: implications for control Health Policy Plan 1998 13 423 432 10.1093/heapol/13.4.423 10346034

[B31] Ostan I Kilimcioglu AA Girginkardesler N Ozyurt BC Limoncu ME Ok UZ Health inequities: lower socio-economic conditions and higher incidences of intestinal parasites BMC Publ Health 2007 7 342 10.1186/1471-2458-7-342 PMC2211470 18042287

[B32] Quihui L Valencia ME Crompton DW Phillips S Hagan P Morales G Role of the employment status and education of mothers in the prevalence of intestinal parasitic infections in mexican rural schoolchildren BMC Publ Health 2006 6 225 10.1186/1471-2458-6-225 PMC1584408 16956417

[B33] Seiskari T Kondrashova A Viskari H Kaila M Haapala AM Aittoniemi J Allergic sensitization and microbial load–a comparison between finland and russian karelia Clin Exp Immunol 2007 148 47 52 10.1111/j.1365-2249.2007.03333.x 17302731 PMC1868862

[B34] Amberbir A Medhin G Erku W Alem A Simms R Robinson K Effects of helicobacter pylori, geohelminth infection and selected commensal bacteria on the risk of allergic disease and sensitization in 3-year-old ethiopian children Clin Exp Allergy 2011 41 1422 1430 10.1111/j.1365-2222.2011.03831.x 21831135

[B35] Martino D Prescott S Epigenetics and prenatal influences on asthma and allergic airways disease Chest 2011 139 640 647 10.1378/chest.10-1800 21362650

[B36] Szepfalusi Z Pichler J Elsasser S van DK Ebner C Bernaschek G Transplacental priming of the human immune system with environmental allergens can occur early in gestation J Allergy Clin Immunol 2000 106 530 536 10.1067/mai.2000.108710 10984374

[B37] Metenou S Suguitan AL Jr Long C Leke RG Taylor DW Fetal immune responses to plasmodium falciparum antigens in a malaria-endemic region of cameroon J Immunol 2007 178 2770 2777 17312120 10.4049/jimmunol.178.5.2770

[B38] Bal MS Mandal NN DAS MK Kar SK Sarangi SS Beuria MK Transplacental transfer of filarial antigens from wuchereria bancrofti-infected mothers to their offspring Parasitology 2010 137 669 673 10.1017/S0031182009991478 19849889

[B39] Eloi-Santos SM Novato-Silva E Maselli VM Gazzinelli G Colley DG Correa-Oliveira R Idiotypic sensitization in utero of children born to mothers with schistosomiasis or Chagas’ disease J Clin Invest 1989 84 1028 1031 10.1172/JCI114225 2503542 PMC329752

[B40] Pit DS Polderman AM Schulz-Key H Soboslay PT Prenatal immune priming with helminth infections: parasite-specific cellular reactivity and Th1 and Th2 cytokine responses in neonates Allergy 2000 55 732 739 10.1034/j.1398-9995.2000.00477.x 10955699

[B41] King CL Malhotra I Mungai P Wamachi A Kioko J Ouma JH B cell sensitization to helminthic infection develops in utero in humans J Immunol 1998 160 3578 3584 9531321

[B42] Supali T Djuardi Y Wibowo H van RR Yazdanbakhsh M Sartono E Relationship between different species of helminths and atopy: a study in a population living in helminth-endemic area in sulawesi, indonesia Int Arch Allergy Immunol 2010 153 388 394 10.1159/000316350 20559005

[B43] Alcantara-Neves NM Badaro SJ dos Santos MC Pontes-de-Carvalho L Barreto ML The presence of serum anti-ascaris lumbricoides IgE antibodies and of trichuris trichiura infection are risk factors for wheezing and/or atopy in preschool-aged brazilian children Respir Res 2010 11 114 10.1186/1465-9921-11-114 20731833 PMC2939601

[B44] Medeiros M Jr Almeida MC Figueiredo JP Atta AM Mendes CM Araujo MI Low frequency of positive skin tests in asthmatic patients infected with schistosoma mansoni exposed to high levels of mite allergens Pediatr Allergy Immunol 2004 15 142 147 10.1046/j.1399-3038.2003.00119.x 15059190

[B45] Santiago HC Bennuru S Boyd A Eberhard M Nutman TB Structural and immunologic cross-reactivity among filarial and mite tropomyosin: implications for the hygiene hypothesis J Allergy Clin Immunol 2011 127 479 486 10.1016/j.jaci.2010.11.007 21185070 PMC3075728

[B46] Mpairwe H Webb EL Muhangi L Ndibazza J Akishule D Nampijja M Anthelminthic treatment during pregnancy is associated with increased risk of infantile eczema: randomised-controlled trial results Pediatr Allergy Immunol 2011 22 305 312 10.1111/j.1399-3038.2010.01122.x 21255083 PMC3130136

